# The largely unnoticed spread of *Clostridioides difficile* PCR ribotype 027 in Germany after 2010

**DOI:** 10.1016/j.infpip.2020.100102

**Published:** 2020-11-03

**Authors:** Vanda Marujo, Mardjan Arvand

**Affiliations:** Robert Koch Institute, Department of Infectious Diseases, Unit for Hospital Hygiene, Infection Prevention and Control, Berlin, Germany

**Keywords:** *Clostridioides difficile*, *C. difficile* infection, PCR ribotype 027, Prevalence, Enhanced surveillance, Germany

## Abstract

In recent decades, incidence and severity of *Clostridioides difficile* infection (CDI) has increased dramatically, coinciding with the emergence of hypervirulent strains such as PCR ribotype 027 (RT027). Data on prevalence of distinct *C. difficile* strains in random CDI cases in Germany are scarce. The aim of this review was to obtain an overview of prevalence and geographical distribution of RT027 among clinical *C. difficile* isolates from random cases in non-outbreak settings in hospitals in Germany. For this purpose, we performed a literature review on reported cases of *C. difficile* RT027 in Germany between 2007 and 2019 in three databases (PubMed, Embase and LIVIVO) and conference proceedings. Studies with selection bias for RT027 (e.g. clinical severity, outbreak reports) were excluded. A total of 304 records were screened, from which 21 were included in this analysis. The nationwide prevalence of RT027 in Germany was <1% prior to 2010 but increased continuously thereafter, reaching 21.7% in 2013. The regional prevalence varied markedly between federal states, higher prevalence was reported from North Rhine-Westphalia (37.4%) and Saxony (31.8%) in 2013-2015. However, data on *C. difficile* RT027 were not available from almost half of the federal states and were scarce at the national level. Our data suggest a remarkable spread of RT027 in Germany during the past decade, which has remained rather unnoticed so far. A national program for molecular surveillance of *C. difficile* is required to monitor the changing epidemiology of CDI and to adjust the prevention and control measures.

## Introduction

*Clostridioides difficile* (*C. difficile*) is a Gram-positive, anaerobic, spore-forming, toxin-producing bacillus [1]. The bacterium is considered the most frequent cause of antibiotic-associated colitis and healthcare-acquired diarrhoea in developed countries [2]. The disease spectrum is wide and ranges from mild diarrhoea to severe infection [3]. *C. difficile* pathogenicity is principally mediated by two exotoxins: toxin A (TcdA) and toxin B (TcdB) [4]. *C. difficile* infection (CDI) primarily affects elderly patients with comorbidities and recent exposure to antibiotics, thereby having major clinical impact on this patient group. Other risk factors for CDI include a compromised immune system, recent stay in a hospital or long-term care facility, and use of acid-suppressive medications [5]. Although CDI is usually associated with hospitalisation, community-acquired CDI have been gaining relevance in recent years [6].

In Germany, CDI was identified as the fourth most commonly diagnosed healthcare-associated infection (HAI) in the national point prevalence survey (PPS) of HAI and antimicrobial use in acute care hospitals in 2016, accounting for 10.0 % of all HAI in the participating hospitals [7]. This rate was twice as high as the average rate (4.8 %) of all countries from the European Union (EU) and European Economic Area (EEA) participating in the EU-wide PPS 2016–2017 organised by the European Centre for Disease Prevention and Control (ECDC) [8]. Furthermore, the prevalence of CDI in German hospitals participating in the PPS in 2016 was significantly higher than the prevalence in 2011 (0.48% vs. 0.34% [7]. As to the burden of HAI in Europe, Cassini *et al.* estimated a median of 1.7 disability-adjusted life years (DALYs) per case of CDI, which is higher than the burden of other HAI such as urinary tract infection, and almost as high as that of pneumonia in this study [9].

The global increase in incidence and severity of CDI over the last decade is linked to the emergence of certain lineages, including the epidemic PCR ribotype 027 [10]. RT027 has been reported as the most predominant PCR ribotype in Canada, the United States and some countries in Central and South America [[10], [11], [12]]. In Europe, outbreaks of RT027 were reported from different countries mostly in 2005–2007 but also in the following years [[13], [14], [15], [16], [17]]. Studies on *C. difficile* epidemiology have revealed an inhomogeneous distribution of RT027 between different European regions [[18], [19], [20], [21], [22]]. The proportion of PCR ribotype 027 isolates correlated with the incidence rate in some studies [23]. Rapid emergence and transcontinental spread of RT027 strains occurred through at least two distinct fluoroquinolone-resistant lineages [24,25]. Moreover, it has been postulated that RT027 global dissemination is still ongoing [26].

In Germany, a hospital outbreak of the *C. difficile* RT027 was reported in 2007 in the south-western state of Rhineland-Palatinate [14]. Mandatory reporting of severe cases of CDI (including detection of RT027 isolates) was introduced as a consequence thereafter [27]. However, since an infection by RT027 does not indicate a severe clinical course *per se,* and because ribotyping data were not available from most cases of CDI, this criterion was later removed in 2016 [28]. According to the German Infection Protection Act (Infektionsschutzgesetz), severe CDI and CDI outbreaks are subject to mandatory reporting, but strain characterisation is not compulsory. Since 2007 severe cases and/or outbreaks of RT027 have been reported from different geographical regions of Germany [29,30]. However, there is no nationwide enhanced surveillance program in place to analyse the circulating strains and ribotypes. The epidemiology of CDI and the prevalence and distribution of RT027 in particular are therefore not well-understood. The aim of this study was to assess published data on the prevalence, distribution, and temporal evolution of *C. difficile* RT027 in Germany since 2007.

## Methods

### Search strategy

We searched electronic databases (PubMed, EMBASE and LIVIVO) using a combination of controlled vocabulary and free text terms (*Clostridium difficile*, *Clostridioides difficil*e, ribotyping, typing, prevalence, occurrence, Germany) for articles published between 2007 and February 2019 with no language restrictions (see supplementary online material for further details). Conference proceedings were also checked for additional studies.

### Eligibility criteria

- Inclusion criteria:
  • Publications reporting molecular typing results for *C. difficile* isolates obtained from patients with the diagnosis of CDI in hospitals or ambulant settings in Germany;
  • Observational studies, single centre or multicentre, published between 2007 and February 2019.
- Exclusion criteria:
  • Studies reporting data on outbreaks (either exclusively or mixed);
  • Studies with overrepresentation of severe disease and/or mandatorily reported cases;
  • Studies reporting data from asymptomatic individuals.

### Data collection and analysis

**Figure 1:**
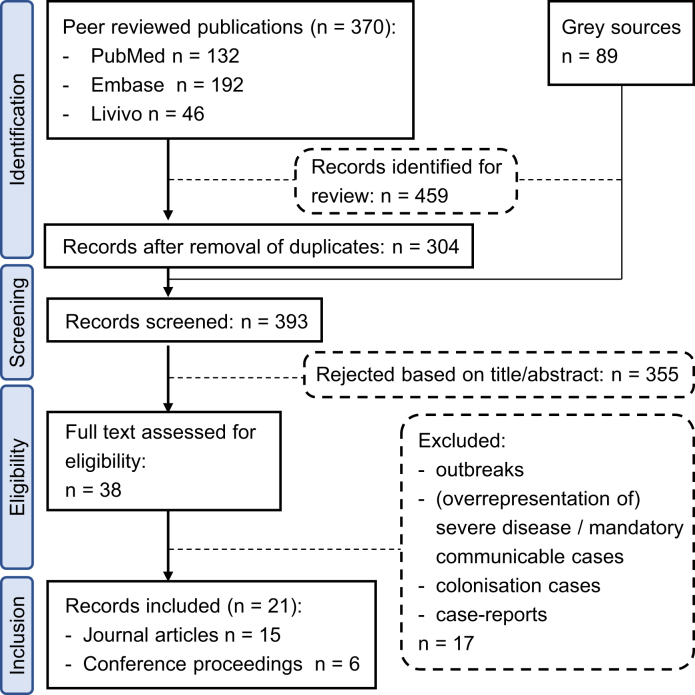
Flow diagram of studies included in this analysis.

We scanned the titles and abstracts of all initially identified publications. When this was insufficient to rule out eligibility, the full text was obtained. The flow diagram of the study selection process is shown in Figure 1. The following data were retrieved from each study: geographical region, time and duration of the study, total number of isolates with ribotyping results, and total number or percentage of identified RT027 isolates. Study authors were contacted for additional information. When data for the same region and time period were reported in more than one publication, peer-reviewed scientific publications were preferred over conference proceedings.

### Statistical analysis

The trend of the RT027 prevalence in Germany between 2005 and 2017 (study time of the included publications) was analysed by a Mann-Kendall test [31] using the Python “mkt” module [32]. The alternative hypothesis of existence of a monotonic upwards trend was tested. For years with RT027 prevalence reported by multiple studies, the mean prevalence of each year, weighted by number of isolates analysed, was used.

## Results

### Included studies

The literature search identified 459 articles and abstracts from conference proceedings, 21 of which fulfilled the inclusion criteria (15 peer-reviewed journal papers and six abstracts) (Figure 1). From the 21 eligible publications, 15 reported regional or local (single centre) data. Among these, six studies analysed data from 2007 until 2010, whilst nine described data between 2011 and 2017. In addition, we found six publications (corresponding to five studies) that examined national data, mainly in the framework of pan-European investigations such as the ClosER or EUCLID study [[19], [20], [21],^33^].

### Distribution and prevalence of C. difficile RT027 at the regional level

**Figure 2:**
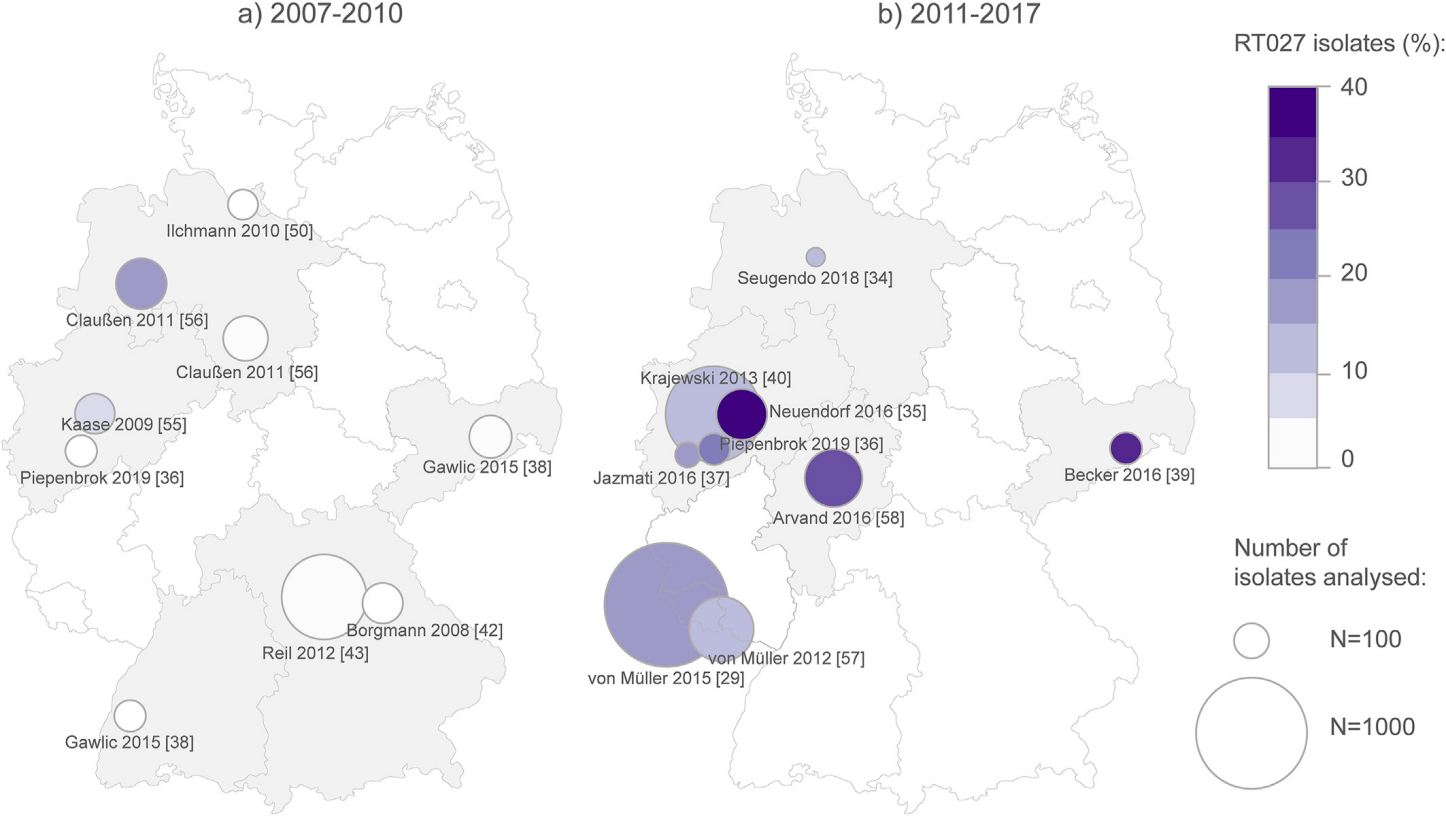
Geographical distribution and prevalence of *C. difficile* RT027 in Germany in different time periods: a) 2007–2010 and b) 2011–2017 (date of collection of isolates). Marked in grey are federal states from which data is available. The circles illustrate occurrence of RT027. The circle size is proportional to the number of isolates analysed, while the colour reflects the proportion of RT027 among typed isolates. Studies are identified by author name and publication year (see Table I).

In the regional/local studies, the proportion of RT027 among all *C. difficile* isolates varied markedly between different regions and different time periods, ranging from zero to 37.4% (Table I). While the prevalence of RT027 was generally low in regional/local studies between 2007 and 2010 (mean = 3.3%), it increased markedly to a mean value of 21.3% in the following years until 2017. Between 2011 and 2017 the prevalence of RT027 ranged between a minimum of 10.3% in Lower Saxony [34] and a maximum of 37.4% in Düsseldorf [35]. A relatively high prevalence was reported mainly from south-west and central Germany, the highest rate being 37.4% reported from Düsseldorf in the State of North Rhine-Westphalia. However, ribotyping data were not available from many regions, particularly from north, north-east and south Germany. Data from different time points were rarely available from the same region or centre. Yet, the few available publications showed a steady increase in prevalence of RT027 over time, i.e. in Cologne from 0% in 2007 to 17.3% in 2014–2015 and 21.3% in 2017 [36,37], in Dresden from 1.4% in 2007–2009 to 31.8% in 2014–2015 [38,39], and in Düsseldorf, from 13.7% in 2010–2012 to 37.4% in 2013–2014 [35,40] (Figure 2).

**Table I:** Regional distribution and prevalence of *C. difficile* PCR ribotype 027 in Germany, 2006–2017

First author	Publication year	Geographical scope	Time of study	RT027 (n/N)	RT027 (%)
Borgmann [42]	2008	Bavaria (north)	2006–2007	0/135	0.0
Piepenbrock [36]	2019	Cologne	2007	0/80	0.0
Gawlic [38]	2015	Freiburg	2007–2009	0/80	0.0
Gawlic [38]	2015	Dresden	2007–2009	2/147	1.4
Ilchmann [50]	2010	Hamburg	2008	0/73	0.0
Kaase [55]	2009	Bochum	2008	9/130	6.9
Reil [43]	2012	Bavaria (north)	2009	27/587	4.6
Claußen [56]	2011	Lower Saxony (southwest)	2009–2010	35/212	16.5
Claußen [56]	2011	Lower Saxony (south)	2010	1/163	0.6
Krajewski [40]	2013	Düsseldorf	2010–2012	103/750	13.7
von Müller [57]	2012	Saarland	2011–2012	50/338	14.8
von Müller [29]	2015	Saarland	2008–2013	231/1 253	18.4
Arvand [58]	2016	Hesse	2011–2014	73/270	27.0
Seugendo [34]	2018	Lower Saxony	2013–2014	3/29	10.3
Neuendorf [35]	2016	Düsseldorf	2013–2014	79/211	37.4
Becker [39]	2016	Dresden	2014–2015	27/85	31.8
Jazmati [37]	2016	Cologne	2014–2015	9/52	17.3
Piepenbrock [36]	2019	Cologne	2017	17/80	21.3

n - number of analysed isolates identified as RT027; N - total number of isolates analysed.

### Distribution and prevalence of C. difficile RT027 at the national level

**Figure 3:**
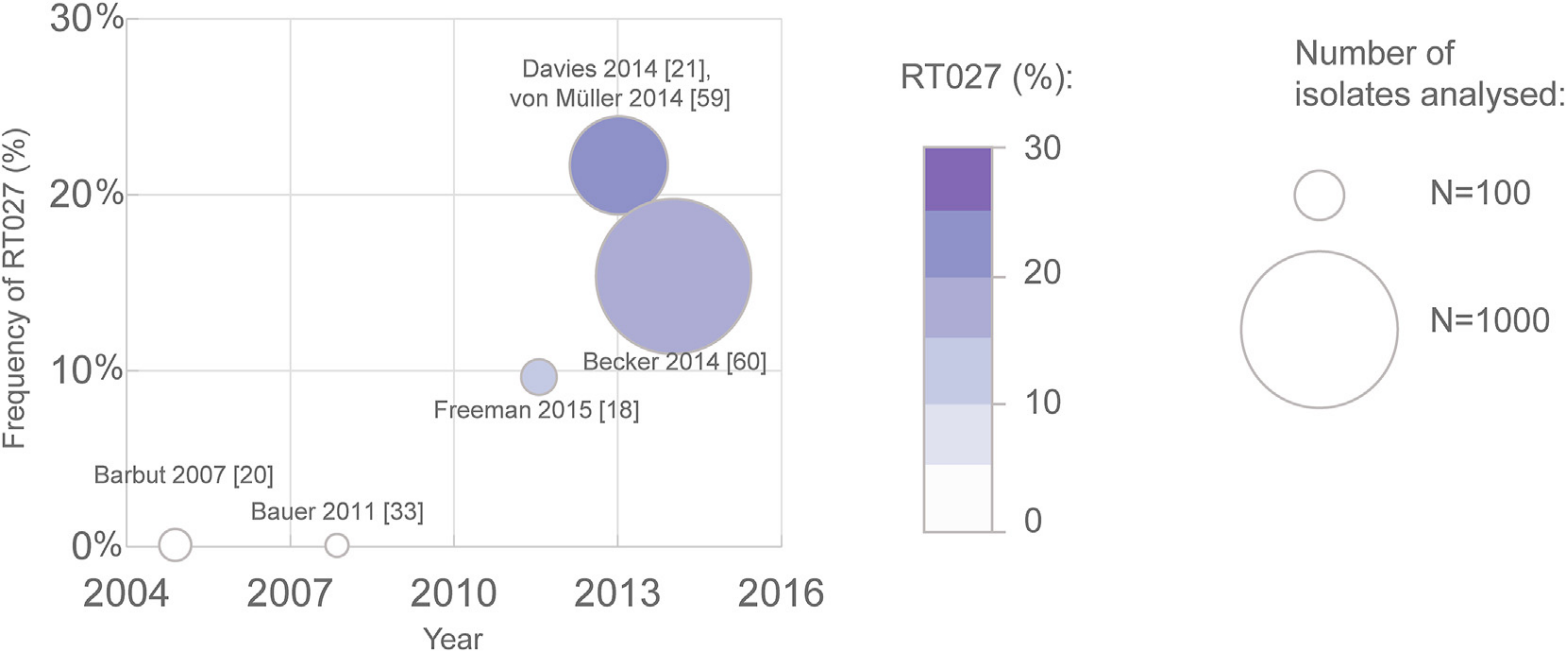
Prevalence of *C. difficile* RT027 in Germany in 2005–2014 (date of collection of isolates). The circles illustrate occurrence of RT027. The circle size is proportional to the number of isolates analysed, while the colour reflects the proportion of RT027 among typed isolates. Studies are identified by author name and publication year (see Table II).

Data were scarce on the national level (Table II). Four out of five national-level studies were performed in the context of pan-European investigations, e.g. the EUCLID study [21]. Whereas no RT027 cases were detected among isolates collected in 2005 and 2008, RT027 accounted for 9.6% of German isolates in 2011–2012, and for 21.7% and 15.8% in studies from 2013 and 2014, respectively (Figure 3).

**Table II:** National prevalence of *C. difficile* PCR ribotype 027 in Germany, 2005–2014

First author	Publication year	Geographical scope	Time of study	RT027 (n/N)	RT027 (%)
Barbut [20]	2007	Not specified	2005	0/42	0.0
Bauer [33]	2011	Not specified	2008	0/22	0.0
Freeman [18]	2015	Not specified (3 sites)	2011–2012	5/52	9.6
Davies [21], von Müller [59]	2014	All states	2013	86/396	21.7
Becker [60]	2014	Several states	2014	156/988	15.8

n –number of analysed isolates identified as RT027; N - total number of isolates analysed.

### Prevalence of other C. difficile ribotypes

Altogether, 30 different PCR ribotypes were reported between 2005 and 2017 (Tables S1-S6 of the supplementary online material). Among these ribotypes, those with the highest frequency were RT001 (23.0%), RT027 (14.8%), and RT014 (6.9%).

### Statistical analysis

Statistical analysis revealed a consistent increase in RT027 prevalence over time (*p*=0.0002). On the contrary, the prevalence of the other two most frequently detected ribotypes, RT001 and RT014, did not increase during the study period (*p*=0.759 and *p*=0.241, respectively).

## Discussion

### Key findings

Our study provides the first systematic overview of the prevalence and distribution of *C. difficile* RT027 in Germany in a non-epidemic setting primarily in hospitals in Germany. It identified a remarkable and largely unperceived increase and regional spread of RT027 during the past decade, reaching prevalence rates of >30% in different federal states in 2014–15.

The prevalence was higher in some central regions and in the south-west; however, there was a data gap especially in north, north-east and south Germany. Regionally, ribotyping data were not available from approximately half of the federal states. Among those federal states with available data, the highest prevalence was reported from North Rhine-Westphalia in 2013–2014 and Saxony in 2014–2015, with 37.4% and 31.8%, respectively [35,39]. These findings suggest that RT027 has become endemic in many hospitals in different regions of Germany.

In this work we chose to exclude studies reporting data on outbreaks or severe clinical manifestation in order to avoid selection bias towards a possible overrepresentation of hypervirulent strains. Interestingly, our results focusing on random cases and non-outbreak settings are in line with the results of the National Advisory Laboratory for *C. difficile*, which showed that RT027 was the second most prevalent ribotype among isolates submitted there between 2011 and 2013 for ribotyping, mainly due to severe clinical disease, recurrence or outbreaks [29].

In our study, we found a remarkable disparity with regard to the prevalence of RT027 between different regions and even within the same region. Data from the same region or centre to different time points were rarely available. However, the few available publications revealed a steadily increasing prevalence of RT027 over time. This finding points towards possible inter- and intra-hospital transmission in some cases. This is in line with previous observations that fluoroquinolone-resistant RT027 strains were imported into Germany at least four times, and that RT027 had been widely disseminated across multiple federal states before the first outbreak was noted in 2007 in south-west Germany [25]. This is in turn in accordance with the data of Eyre *et al.* suggesting within-country clustering of RT027 in Germany [41]. Furthermore, these authors showed that RT027 clustering also occurred regionally and within-hospitals and found a strong association between clustering and fluoroquinolone resistance [41].

The data included in this review are mainly obtained from hospitalised patients. All studies included either exclusively inpatient samples or a mix of in- and outpatient samples, with the majority being inpatients. However, only two studies provided clear information on this topic [42,43], both of which including > 80% inpatient samples. It is therefore difficult to draw conclusions on the prevalence and circulation of RT027 in the community setting in Germany, yet it would be interesting to assess this in further studies.

### Implications

Standardised CDI diagnostics is one of the key components of surveillance. The EUCLID study provided evidence that CDI was underdiagnosed in Europe, probably because of low awareness of the indications and requirements for *C. difficile* testing among physicians [21]. The high prevalence of CDI in Germany suggests the need for an increased awareness of clinicians of CDI as well as of the spread of epidemic strains in German hospitals. Since testing for *C. difficile* is not routinely included in the laboratory diagnostic workup for diarrhoeal samples of hospitalized patients in Germany, a test for *C. difficile* needs to be actively requested when the disease is clinically suspected. In addition, the detection of clusters and outbreaks requires established surveillance, including knowledge of the department-specific frequency of CDI as well as of the prevalence of different ribotypes (e.g. by using the enhanced surveillance protocol). According to the German Infection Protection Act and the German national guidelines on outbreak management and on prevention and control of CDI, a nosocomial outbreak is defined as two or more nosocomial infections for which an epidemiological link is likely or suspected [44]. In case of high CDI incidence or suspicion of nosocomial outbreak, typing of isolates is recommended [45].

Since microbiological culture and characterisation of *C. difficile* strains are not performed on a regular or systematic basis in Germany, there is currently a lack of information on molecular epidemiology of CDI in the country. At the European level, a CDI surveillance system was in place in 20 countries in 2017 and 21 countries (70% of all EU/EEA countries) participated in ECDC-coordinated CDI surveillance in acute care hospitals in 2016, using a common protocol and thus allowing data comparison [46,47]. The ECDC surveillance protocol allows three options for data collection: ‘minimal’ (aggregated hospital data); ‘light’ (including patient data such as mortality) or ‘enhanced’ (including case-based microbiological data) [48]. Germany did not participate in 2016, but tested the pilot protocol in 2013 [23]. Some countries (e.g. Finland, the Netherlands, and the United Kingdom) performed enhanced surveillance of CDI. Enhanced surveillance supports early recognition of outbreaks due to new *C. difficile* ribotypes and the distribution of certain ribotypes in specific populations, and also a faster identification of new strains associated with increased morbidity or mortality, facilitating initiation of appropriate control measures [47]. Appropriate microbiological diagnosis and participation in epidemiological surveillance are two pillars of the prevention and control of CDI in healthcare facilities [49]. In conclusion, it would be desirable for Germany to carry out enhanced surveillance in the future.

### Limitations

Our study also has some limitations. Firstly, the included studies were heterogeneous with respect to the study design, sampling strategy and sample size. In addition, not all studies aimed to evaluate rates of *C. difficile* infection and/or classify strains; some were designed to investigate diagnostic tools [35,38] or antimicrobial resistance [18,50]. Nonetheless, these studies were included whenever it was possible to assess the rate of RT027 CDI. Moreover, information on indication for *C. difficile* testing, definition of diarrhoea or classification of diarrheic stools at the laboratory level was not available in all studies included in this review. With regard to sampling, although *C. difficile* isolates were collected as part of routine microbiological diagnostics in most studies, a few studies also included samples submitted for typing due to severity of disease. Finally, the sample size was small in most studies, with a median of 135 and a range of 22–1253. Thus, the representativeness of the results is compromised.

Furthermore, different typing methods were used in different studies, which may have had an impact on the ribotyping results [51]. For example, some ribotypes closely related to RT027 (for instance, RT176) may be difficult to distinguish and hence be falsely classified [52,53]. In addition, inter-laboratory standardization is difficult to achieve. Although PCR ribotyping was indeed the most frequent typing method, in line with the European harmonized diagnostic procedures [54], even with this method it is difficult to compare data between laboratories without reference strains [51].

On the other hand, our review has likely identified all available studies on the topic, since we searched three different databases with broad search criteria, complemented by the inclusion of grey literature. We further tried to avoid bias by defining inclusion criteria clearly and thoroughly. Secondly, in the case of studies with partially overlapping investigation periods, only the study with the longer duration (and therefore more samples) was included in order to avoid overrepresentation data.

## Conclusions

In summary, this paper provides the first systematic overview of the prevalence and temporal development of *C. difficile* RT027 among random CDI cases in non-epidemic settings in German hospitals. The results of our study further emphasize the need for a nationwide program for enhanced surveillance of CDI and to monitor the changing epidemiology especially with regard to the nosocomial spread of epidemic strains. These data may help to better adjust the prevention and control strategies in order to reduce the incidence of CDI in Germany.

## Author contributions

Writing – Original Draft: V.M.; Writing – Review & Editing: V.M. and M.A.; Conceptualization – M.A.; Methodology – V.M.; Formal Analysis – V.M.; Investigation: V.M.; Data curation – V.M. and M.A.; Visualization – V.M.; Supervision – M.A.
