# Supplementary material for: The largely unnoticed spread of Clostridioides difficile PCR ribotype 027 in Germany after 2010
Source: Infect Prev Pract. 2020 Nov 3;2(4):100102. doi: 10.1016/j.infpip.2020.100102 (PMC8336157; doi:10.1016/j.infpip.2020.100102)
Supplement: Multimedia component 1 [file mmc1.docx]

**Supplementary Material**

This material contains the search strategy and supplementary tables as supporting information alongside the article ‘The largely unnoticed spread of *Clostridioides difficile* PCR ribotype 027 in Germany after 2010’.

**Search Strategy:**

**Search strategy in PubMed**

(01 OR 02) AND 03 AND (04 OR 05 OR 06 OR 07) AND 08 AND 09, where:

1. “clostridium difficile” [Title/Abstract]
2. “clostridioides difficile” [Title/Abstract])
3. Germany
4. ribotyping
5. typing
6. prevalence
7. occurrence
8. “2007/01/01” [PDat] : “2019/02/28” [PDat]
9. Humans [Mesh]

**Search strategy in Livivo**

(01 OR 02) AND (03 OR 04) AND (05 OR 06 OR 07 OR 08 OR 09) AND 10 AND 11, where:

1. TI=clostridium difficile
2. TI=clostridioides difficile
3. Germany
4. Deutschland
5. ribotyping
6. typing
7. prevalence
8. Prävalenz
9. occurrence
10. PY=2007:2019
11. Filter Subject: “Medizin”, “Gesundheit”

**Search strategy in Embase**

(01 OR 02) AND (03 OR 04) AND (05 OR 06 OR 07 OR 08 OR 09 OR 10) AND 11 AND 12, where:

1. 'clostridium difficile':ab,ti
2. 'clostridioides difficile':ab,ti
3. 'Germany'/exp
4. Germany
5. 'ribotyping'/exp
6. ribotyping
7. typing
8. 'prevalence'/exp
9. prevalence
10. occurrence
11. [2007-2019]/py
12. 'human'/de

**Supplementary Tables**

Supplementary Table S1. Ribotypes identified in the articles included in this study, with the count of the number of articles that found a positive identification, and the mean relative frequency taking into account all articles.

| Ribotype | Count num. articles with n>0 | Mean % = Sum(n)/Sum(N)*100 |
| --- | --- | --- |
| 001 | 13 | 23.0 |
| 002 | 8 | 1.0 |
| 003 | 2 | 0.1 |
| 005 | 1 | 0.0 |
| 010 | 3 | 0.4 |
| 012 | 3 | 0.2 |
| 014 | 14 | 6.9 |
| 015 | 4 | 0.4 |
| 017 | 1 | 0.0 |
| 020 | 3 | 0.1 |
| 023 | 2 | 0.2 |
| 027 | 17 | 14.8 |
| 029 | 2 | 0.2 |
| 031 | 1 | 0.5 |
| 032 | 1 | 0.0 |
| 042 | 2 | 0.0 |
| 045 | 1 | 0.0 |
| 046 | 4 | 0.6 |
| 049 | 1 | 0.0 |
| 053 | 1 | 1.3 |
| 056 | 1 | 0.1 |
| 066 | 1 | 0.0 |
| 077 | 1 | 0.3 |
| 078 | 11 | 1.2 |
| 081 | 3 | 0.1 |
| 087 | 1 | 0.0 |
| 095 | 1 | 0.1 |
| 126 | 3 | 0.3 |
| 140 | 1 | 0.2 |
| 149 | 1 | 0.1 |

Supplementary Table S2. Ribotypes identified in the studies included in this study, with their total number of isolates and relative frequency, for ribotypes 001, 002, 003, 005, 010 and 012.

| First author | Time of study | N | 001 | | 002 | | 003 | | 005 | | 010 | | 012 | |
| --- | --- | --- | --- | --- | --- | --- | --- | --- | --- | --- | --- | --- | --- | --- |
|  |  |  | n | % | n | % | n | % | n | % | n | % | n | % |
| Arvand | 2011-2014 | 270 | 84 | 31.1 | 9 | 3,3 | 0 | 0.0 | 0 | 0.0 | 0 | 0.0 | 0 | 0.0 |
| Barbut | 2005 | 42 | 0 | 0.0 | 0 | 0.0 | 0 | 0.0 | 0 | 0.0 | 0 | 0.0 | 0 | 0.0 |
| Bauer | 2008 | 22 | 0 | 0.0 | 0 | 0.0 | 0 | 0.0 | 0 | 0.0 | 0 | 0.0 | 0 | 0.0 |
| Becker | 2014 | 988 | 0 | 0.0 | 0 | 0.0 | 0 | 0.0 | 0 | 0.0 | 0 | 0.0 | 0 | 0.0 |
| Becker | 2014-2015 | 85 | 0 | 0.0 | 0 | 0.0 | 0 | 0.0 | 0 | 0.0 | 0 | 0.0 | 0 | 0.0 |
| Borgmann | 2006-2007 | 135 | 11 | 8.1 | 0 | 0.0 | 0 | 0.0 | 0 | 0.0 | 0 | 0.0 | 0 | 0.0 |
| Claußen | 2010 | 163 | 130 | 79.8 | 0 | 0.0 | 0 | 0.0 | 0 | 0.0 | 0 | 0.0 | 0 | 0.0 |
| Claußen | 2009-2010 | 212 |  | 42.0 | 0 | 0.0 | 0 | 0.0 | 0 | 0.0 | 0 | 0.0 | 0 | 0.0 |
| Davies / von Müller | 2013 | 396 | 76 | 19.1 | 15 | 3.8 | 0 | 0.0 | 0 | 0.0 | 0 | 0.0 | 0 | 0.0 |
| Freeman | 2011-2012 | 52 | 12 | 23.1 | 3 | 5.8 | 0 | 0.0 | 0 | 0.0 | 0 | 0.0 | 0 | 0.0 |
| Gawlic | 2007-2009 | 147 | 0 | 0.0 | 0 | 0.0 | 0 | 0.0 | 0 | 0.0 | 0 | 0.0 | 0 | 0.0 |
| Gawlic | 2007-2009 | 80 | 0 | 0.0 | 0 | 0.0 | 0 | 0.0 | 0 | 0.0 | 0 | 0.0 | 0 | 0.0 |
| Ilchmann | 2008 | 73 | 44 | 60.3 | 0 | 0.0 | 3 | 4.1 | 0 | 0.0 | 1 | 1.4 | 1 | 1.4 |
| Jazmati | 2014-2015 | 52 | 0 | 0.0 | 0 | 0.0 | 0 | 0.0 | 0 | 0.0 | 0 | 0.0 | 0 | 0.0 |
| Kaase | 2008 | 130 | 0 | 0.0 | 0 | 0.0 | 0 | 0.0 | 0 | 0.0 | 0 | 0.0 | 0 | 0.0 |
| Krajewski | 2010-2012 | 750 | 257 | 34.3 | 0 | 0.0 | 0 | 0.0 | 0 | 0.0 | 0 | 0.0 | 0 | 0.0 |
| Neuendorf | 2013-2014 | 211 | 0 | 0.0 | 0 | 0.0 | 0 | 0.0 | 0 | 0.0 | 0 | 0.0 | 0 | 0.0 |
| Piepenbrock | 2017 | 80 | 3 | 3.8 | 3 | 3.8 | 0 | 0.0 | 0 | 0.0 | 0 | 0.0 | 0 | 0.0 |
| Piepenbrock | 2007 | 80 | 15 | 18.8 | 7 | 8.8 | 0 | 0.0 | 0 | 0.0 | 0 | 0.0 | 0 | 0.0 |
| Reil | 2009 | 587 | 423 | 72.1 | 8 | 1.4 | 3 | 0.5 | 3 | 0.5 | 0 | 0.0 | 6 | 1.0 |
| Seugendo | 2013-2014 | 29 | 18 | 62.1 | 4 | 13.8 | 0 | 0.0 | 0 | 0.0 | 1 | 3.4 | 0 | 0.0 |
| von Müller | 2011-2012 | 338 | 62 | 18.3 | 11 | 3.3 | 0 | 0.0 | 0 | 0.0 | 21 | 6.2 | 8 | 2.4 |
| von Müller | 2008-2013 | 1253 | 283 | 22.6 | 0 | 0.0 | 0 | 0.0 | 0 | 0.0 | 0 | 0.0 | 0 | 0.0 |

Supplementary Table S3. Ribotypes identified in the studies included in this study, with their total number of isolates and relative frequency, for ribotypes 014, 015, 017, 020, 023 and 027.

| First author | Time of study | N | 014 | | 015 | | 017 | | 020 | | 023 | | 027 | |
| --- | --- | --- | --- | --- | --- | --- | --- | --- | --- | --- | --- | --- | --- | --- |
|  |  |  | n | % | n | % | n | % | n | % | n | % | n | % |
| Arvand | 2011-2014 | 270 | 26 | 9.6 | 0 | 0.0 | 0 | 0.0 | 0 | 0.0 | 0 | 0.0 | 73 | 27.0 |
| Barbut | 2005 | 42 | 0 | 0.0 | 0 | 0.0 | 0 | 0.0 | 0 | 0.0 | 0 | 0.0 | 0 | 0.0 |
| Bauer | 2008 | 22 | 0 | 0.0 | 0 | 0.0 | 0 | 0.0 | 0 | 0.0 | 0 | 0.0 | 0 | 0.0 |
| Becker | 2014 | 988 | 0 | 0.0 | 0 | 0.0 | 0 | 0.0 | 0 | 0.0 | 0 | 0.0 | 156 | 15.8 |
| Becker | 2014-2015 | 85 | 0 | 0.0 | 0 | 0.0 | 0 | 0.0 | 0 | 0.0 | 0 | 0.0 | 27 | 31.8 |
| Borgmann | 2006-2007 | 135 | 1 | 0.7 | 1 | 0.7 | 0 | 0.0 | 0 | 0.0 | 0 | 0.0 | 0 | 0.0 |
| Claußen | 2010 | 163 | 9 | 5.5 | 0 | 0.0 | 0 | 0.0 | 0 | 0.0 | 0 | 0.0 | 1 | 0.6 |
| Claußen | 2009-2010 | 212 | 13 | 6.1 | 0 | 0.0 | 0 | 0.0 | 0 | 0.0 | 0 | 0.0 | 35 | 16.5 |
| Davies / von Müller | 2013 | 396 | 40 | 10.1 | 11 | 2.8 | 0 | 0.0 | 0 | 0.0 | 0 | 0.0 | 86 | 21.7 |
| Freeman | 2011-2012 | 52 | 5 | 9.6 | 0 | 0.0 | 0 | 0.0 | 0 | 0.0 | 0 | 0.0 | 5 | 9.6 |
| Gawlic | 2007-2009 | 147 | 0 | 0.0 | 0 | 0.0 | 0 | 0.0 | 0 | 0.0 | 0 | 0.0 | 2 | 1.4 |
| Gawlic | 2007-2009 | 80 | 0 | 0.0 | 0 | 0.0 | 0 | 0.0 | 0 | 0.0 | 0 | 0.0 | 0 | 0.0 |
| Ilchmann | 2008 | 73 | 8 | 11.0 | 1 | 1.4 | 0 | 0.0 | 1 | 1.4 | 1 | 1.4 | 0 | 0.0 |
| Jazmati | 2014-2015 | 52 | 0 | 0.0 | 0 | 0.0 | 0 | 0.0 | 0 | 0.0 | 0 | 0.0 | 9 | 17.3 |
| Kaase | 2008 | 130 | 0 | 0.0 | 0 | 0.0 | 0 | 0.0 | 0 | 0.0 | 0 | 0.0 | 9 | 6.9 |
| Krajewski | 2010-2012 | 750 | 85 | 11.3 | 0 | 0.0 | 0 | 0.0 | 0 | 0.0 | 0 | 0.0 | 103 | 13.7 |
| Neuendorf | 2013-2014 | 211 | 0 | 0.0 | 0 | 0.0 | 0 | 0.0 | 0 | 0.0 | 0 | 0.0 | 79 | 37.4 |
| Piepenbrock | 2017 | 80 | 8 | 10.0 | 0 | 0.0 | 0 | 0.0 | 5 | 6.3 | 0 | 0.0 | 17 | 21.3 |
| Piepenbrock | 2007 | 80 | 3 | 3.8 | 0 | 0.0 | 0 | 0.0 | 2 | 2.5 | 0 | 0.0 | 0 | 0.0 |
| Reil | 2009 | 587 | 4 | 0.7 | 9 | 1.5 | 0 | 0.0 | 0 | 0.0 | 14 | 2.4 | 27 | 4.6 |
| Seugendo | 2013-2014 | 29 | 3 | 10.3 | 0 | 0.0 | 0 | 0.0 | 0 | 0.0 | 0 | 0.0 | 3 | 10.3 |
| von Müller | 2011-2012 | 338 | 55 | 16.3 | 0 | 0.0 | 2 | 0.6 | 0 | 0.0 | 0 | 0.0 | 50 | 14.8 |
| von Müller | 2008-2013 | 1253 | 168 | 13.4 | 0 | 0.0 | 0 | 0.0 | 0 | 0.0 | 0 | 0.0 | 231 | 18.4 |

Supplementary Table S4. Ribotypes identified in the studies included in this study, with their total number of isolates and relative frequency, for ribotypes 029, 031, 032, 042, 045 and 046.

| First author | Time of study | N | 029 | | 031 | | 032 | | 042 | | 045 | | 046 | |
| --- | --- | --- | --- | --- | --- | --- | --- | --- | --- | --- | --- | --- | --- | --- |
|  |  |  | n | % | n | % | n | % | n | % | n | % | n | % |
| Arvand | 2011-2014 | 270 | 6 | 2.2 | 0 | 0.0 | 0 | 0.0 | 0 | 0.0 | 0 | 0.0 | 0 | 0.0 |
| Barbut | 2005 | 42 | 0 | 0.0 | 0 | 0.0 | 0 | 0.0 | 0 | 0.0 | 0 | 0.0 | 0 | 0.0 |
| Bauer | 2008 | 22 | 0 | 0.0 | 0 | 0.0 | 0 | 0.0 | 0 | 0.0 | 0 | 0.0 | 0 | 0.0 |
| Becker | 2014 | 988 | 0 | 0.0 | 0 | 0.0 | 0 | 0.0 | 0 | 0.0 | 0 | 0.0 | 0 | 0.0 |
| Becker | 2014-2015 | 85 | 0 | 0.0 | 0 | 0.0 | 0 | 0.0 | 0 | 0.0 | 0 | 0.0 | 0 | 0.0 |
| Borgmann | 2006-2007 | 135 | 0 | 0.0 | 0 | 0.0 | 0 | 0.0 | 1.0 | 0.7 | 0 | 0.0 | 0 | 0.0 |
| Claußen | 2010 | 163 | 0 | 0.0 | 0 | 0.0 | 0 | 0.0 | 0 | 0.0 | 0 | 0.0 | 0 | 0.0 |
| Claußen | 2009-2010 | 212 | 0 | 0.0 | 0 | 0.0 | 0 | 0.0 | 0 | 0.0 | 0 | 0.0 | 23 | 10.8 |
| Davies / von Müller | 2013 | 396 | 0 | 0.0 | 0 | 0.0 | 0 | 0.0 | 0 | 0.0 | 0 | 0.0 | 0 | 0.0 |
| Freeman | 2011-2012 | 52 | 0 | 0.0 | 0 | 0.0 | 0 | 0.0 | 0 | 0.0 | 0 | 0.0 | 0 | 0.0 |
| Gawlic | 2007-2009 | 147 | 0 | 0.0 | 0 | 0.0 | 0 | 0.0 | 0 | 0.0 | 0 | 0.0 | 0 | 0.0 |
| Gawlic | 2007-2009 | 80 | 0 | 0.0 | 0 | 0.0 | 0 | 0.0 | 0 | 0.0 | 0 | 0.0 | 0 | 0.0 |
| Ilchmann | 2008 | 73 | 0 | 0.0 | 0 | 0.0 | 1 | 1.4 | 1 | 1.4 | 1 | 1.4 | 1 | 1.4 |
| Jazmati | 2014-2015 | 52 | 0 | 0.0 | 0 | 0.0 | 0 | 0.0 | 0 | 0.0 | 0 | 0.0 | 0 | 0.0 |
| Kaase | 2008 | 130 | 0 | 0.0 | 0 | 0.0 | 0 | 0.0 | 0 | 0.0 | 0 | 0.0 | 0 | 0.0 |
| Krajewski | 2010-2012 | 750 | 0 | 0.0 | 0 | 0.0 | 0 | 0.0 | 0 | 0.0 | 0 | 0.0 | 0 | 0.0 |
| Neuendorf | 2013-2014 | 211 | 0 | 0.0 | 0 | 0.0 | 0 | 0.0 | 0 | 0.0 | 0 | 0.0 | 0 | 0.0 |
| Piepenbrock | 2017 | 80 | 0 | 0.0 | 0 | 0.0 | 0 | 0.0 | 0 | 0.0 | 0 | 0.0 | 0 | 0.0 |
| Piepenbrock | 2007 | 80 | 0 | 0.0 | 0 | 0.0 | 0 | 0.0 | 0 | 0.0 | 0 | 0.0 | 8 | 10.0 |
| Reil | 2009 | 587 | 5 | 0.9 | 0 | 0.0 | 0 | 0.0 | 0 | 0.0 | 0 | 0.0 | 5 | 0.9 |
| Seugendo | 2013-2014 | 29 | 0 | 0.0 | 0 | 0.0 | 0 | 0.0 | 0 | 0.0 | 0 | 0.0 | 0 | 0.0 |
| von Müller | 2011-2012 | 338 | 0 | 0.0 | 28 | 8.3 | 0 | 0.0 | 0 | 0.0 | 0 | 0.0 | 0 | 0.0 |
| von Müller | 2008-2013 | 1253 | 0 | 0.0 | 0 | 0.0 | 0 | 0.0 | 0 | 0.0 | 0 | 0.0 | 0 | 0.0 |

Supplementary Table S5. Ribotypes identified in the studies included in this study, with their total number of isolates and relative frequency, for ribotypes 049, 053, 056, 066, 077 and 078.

| First author | Time of study | N | 049 | | 053 | | 056 | | 066 | | 077 | | 078 | |
| --- | --- | --- | --- | --- | --- | --- | --- | --- | --- | --- | --- | --- | --- | --- |
|  |  |  | n | % | n | % | n | % | n | % | n | % | n | % |
| Arvand | 2011-2014 | 270 | 0 | 0.0 | 0 | 0.0 | 0 | 0.0 | 0 | 0.0 | 0 | 0.0 | 8 | 3.0 |
| Barbut | 2005 | 42 | 0 | 0.0 | 0 | 0.0 | 0 | 0.0 | 0 | 0.0 | 0 | 0.0 | 0 | 0.0 |
| Bauer | 2008 | 22 | 0 | 0.0 | 0 | 0.0 | 0 | 0.0 | 0 | 0.0 | 0 | 0.0 | 0 | 0.0 |
| Becker | 2014 | 988 | 0 | 0.0 | 0 | 0.0 | 0 | 0.0 | 0 | 0.0 | 0 | 0.0 | 0 | 0.0 |
| Becker | 2014-2015 | 85 | 0 | 0.0 | 0 | 0.0 | 0 | 0.0 | 0 | 0.0 | 0 | 0.0 | 0 | 0.0 |
| Borgmann | 2006-2007 | 135 | 1 | 0.7 | 0 | 0.0 | 0 | 0.0 | 0 | 0.0 | 0 | 0.0 | 1 | 0.7 |
| Claußen | 2010 | 163 | 0 | 0.0 | 0 | 0.0 | 0 | 0.0 | 0 | 0.0 | 0 | 0.0 | 6 | 3.7 |
| Claußen | 2009-2010 | 212 | 0 | 0.0 | 0 | 0.0 | 0 | 0.0 | 0 | 0.0 | 0 | 0.0 | 15 | 7.1 |
| Davies / von Müller | 2013 | 396 | 0 | 0.0 | 0 | 0.0 | 0 | 0.0 | 0 | 0.0 | 0 | 0.0 | 10 | 2.6 |
| Freeman | 2011-2012 | 52 | 0 | 0.0 | 0 | 0.0 | 0 | 0.0 | 0 | 0.0 | 0 | 0.0 | 0 | 0.0 |
| Gawlic | 2007-2009 | 147 | 0 | 0.0 | 0 | 0.0 | 0 | 0.0 | 0 | 0.0 | 0 | 0.0 | 0 | 0.0 |
| Gawlic | 2007-2009 | 80 | 0 | 0.0 | 0 | 0.0 | 0 | 0.0 | 0 | 0.0 | 0 | 0.0 | 0 | 0.0 |
| Ilchmann | 2008 | 73 | 0 | 0.0 | 0 | 0.0 | 0 | 0.0 | 1 | 1.4 | 0 | 0.0 | 1 | 1.4 |
| Jazmati | 2014-2015 | 52 | 0 | 0.0 | 0 | 0.0 | 0 | 0.0 | 0 | 0.0 | 0 | 0.0 | 0 | 0.0 |
| Kaase | 2008 | 130 | 0 | 0.0 | 0 | 0.0 | 0 | 0.0 | 0 | 0.0 | 0 | 0.0 | 0 | 0.0 |
| Krajewski | 2010-2012 | 750 | 0 | 0.0 | 78 | 10.4 | 0 | 0.0 | 0 | 0.0 | 0 | 0.0 | 0 | 0.0 |
| Neuendorf | 2013-2014 | 211 | 0 | 0.0 | 0 | 0.0 | 0 | 0.0 | 0 | 0.0 | 0 | 0.0 | 0 | 0.0 |
| Piepenbrock | 2017 | 80 | 0 | 0.0 | 0 | 0.0 | 0 | 0.0 | 0 | 0.0 | 0 | 0.0 | 4 | 5.0 |
| Piepenbrock | 2007 | 80 | 0 | 0.0 | 0 | 0.0 | 0 | 0.0 | 0 | 0.0 | 0 | 0.0 | 3 | 3.8 |
| Reil | 2009 | 587 | 0 | 0.0 | 0 | 0.0 | 6 | 1.0 | 0 | 0.0 | 17 | 2.9 | 10 | 1.7 |
| Seugendo | 2013-2014 | 29 | 0 | 0.0 | 0 | 0.0 | 0 | 0.0 | 0 | 0.0 | 0 | 0.0 | 6 | 20.7 |
| von Müller | 2011-2012 | 338 | 0 | 0.0 | 0 | 0.0 | 0 | 0.0 | 0 | 0.0 | 0 | 0.0 | 8 | 2.4 |
| von Müller | 2008-2013 | 1253 | 0 | 0.0 | 0 | 0.0 | 0 | 0.0 | 0 | 0.0 | 0 | 0.0 | 0 | 0.0 |

Supplementary Table S6. Ribotypes identified in the studies included in this study, with their total number of isolates and relative frequency, for ribotypes 081, 087, 095, 126, 140 and 149.

| First author | Time of study | N | 081 | | 087 | | 095 | | 126 | | 140 | | 149 | |
| --- | --- | --- | --- | --- | --- | --- | --- | --- | --- | --- | --- | --- | --- | --- |
|  |  |  | n | % | n | % | n | % | n | % | n | % | n | % |
| Arvand | 2011-2014 | 270 | 0 | 0.0 | 0 | 0.0 | 0 | 0.0 | 0 | 0.0 | 0 | 0.0 | 0 | 0.0 |
| Barbut | 2005 | 42 | 0 | 0.0 | 0 | 0.0 | 0 | 0.0 | 0 | 0.0 | 0 | 0.0 | 0 | 0.0 |
| Bauer | 2008 | 22 | 0 | 0.0 | 0 | 0.0 | 0 | 0.0 | 0 | 0.0 | 0 | 0.0 | 0 | 0.0 |
| Becker | 2014 | 988 | 0 | 0.0 | 0 | 0.0 | 0 | 0.0 | 0 | 0.0 | 0 | 0.0 | 0 | 0.0 |
| Becker | 2014-2015 | 85 | 0 | 0.0 | 0 | 0.0 | 0 | 0.0 | 0 | 0.0 | 0 | 0.0 | 0 | 0.0 |
| Borgmann | 2006-2007 | 135 | 1 | 0.7 | 0 | 0.0 | 0 | 0.0 | 0 | 0.0 | 0 | 0.0 | 0 | 0.0 |
| Claußen | 2010 | 163 | 0 | 0.0 | 0 | 0.0 | 0 | 0.0 | 0 | 0.0 | 0 | 0.0 | 0 | 0.0 |
| Claußen | 2009-2010 | 212 | 0 | 0.0 | 0 | 0.0 | 0 | 0.0 | 0 | 0.0 | 0 | 0.0 | 0 | 0.0 |
| Davies / von Müller | 2013 | 396 | 0 | 0.0 | 0 | 0.0 | 0 | 0.0 | 0 | 0.0 | 12 | 3.1 | 0 | 0.0 |
| Freeman | 2011-2012 | 52 | 0 | 0.0 | 0 | 0.0 | 0 | 0.0 | 0 | 0.0 | 0 | 0.0 | 0 | 0.0 |
| Gawlic | 2007-2009 | 147 | 0 | 0.0 | 0 | 0.0 | 0 | 0.0 | 0 | 0.0 | 0 | 0.0 | 0 | 0.0 |
| Gawlic | 2007-2009 | 80 | 0 | 0.0 | 0 | 0.0 | 0 | 0.0 | 0 | 0.0 | 0 | 0.0 | 0 | 0.0 |
| Ilchmann | 2008 | 73 | 2 | 2.7 | 1 | 1.4 | 0 | 0.0 | 0 | 0.0 | 0 | 0.0 | 0 | 0.0 |
| Jazmati | 2014-2015 | 52 | 0 | 0.0 | 0 | 0.0 | 0 | 0.0 | 0 | 0.0 | 0 | 0.0 | 0 | 0.0 |
| Kaase | 2008 | 130 | 0 | 0.0 | 0 | 0.0 | 0 | 0.0 | 0 | 0.0 | 0 | 0.0 | 0 | 0.0 |
| Krajewski | 2010-2012 | 750 | 0 | 0.0 | 0 | 0.0 | 0 | 0.0 | 0 | 0.0 | 0 | 0.0 | 0 | 0.0 |
| Neuendorf | 2013-2014 | 211 | 0 | 0.0 | 0 | 0.0 | 0 | 0.0 | 0 | 0.0 | 0 | 0.0 | 0 | 0.0 |
| Piepenbrock | 2017 | 80 | 0 | 0.0 | 0 | 0.0 | 0 | 0.0 | 6 | 7.5 | 0 | 0.0 | 0 | 0.0 |
| Piepenbrock | 2007 | 80 | 0 | 0.0 | 0 | 0.0 | 0 | 0.0 | 3 | 3.8 | 0 | 0.0 | 0 | 0.0 |
| Reil | 2009 | 587 | 4 | 0.7 | 0 | 0.0 | 8 | 1.4 | 11 | 1.9 | 0 | 0.0 | 4 | 0.7 |
| Seugendo | 2013-2014 | 29 | 0 | 0.0 | 0 | 0.0 | 0 | 0.0 | 0 | 0.0 | 0 | 0.0 | 0 | 0.0 |
| von Müller | 2011-2012 | 338 | 0 | 0.0 | 0 | 0.0 | 0 | 0.0 | 0 | 0.0 | 0 | 0.0 | 0 | 0.0 |
| von Müller | 2008-2013 | 1253 | 0 | 0.0 | 0 | 0.0 | 0 | 0.0 | 0 | 0.0 | 0 | 0.0 | 0 | 0.0 |
